# The impacts of health systems financing fragmentation in low- and middle-income countries: a systematic review protocol

**DOI:** 10.1186/s13643-021-01714-5

**Published:** 2021-06-02

**Authors:** Marina Siqueira, Maíra Coube, Christopher Millett, Rudi Rocha, Thomas Hone

**Affiliations:** 1 Institute for Health Policy Studies, IEPS, Itapeva St 286, Conjunto 81-84, Bela Vista, São Paulo, SP 01332-000 Brazil; 2 Public Health Policy Evaluation Unit, Department of Primary Care and Public Health, School of Public Health, Imperial College London, Exhibition Rd, South Kensington, London, SW7 2BU UK

**Keywords:** Health systems, Fragmentation, Financing, Outcomes, LMICs

## Abstract

**Background:**

Health systems are often fragmented in low- and middle-income countries (LMICs). This can increase inefficiencies and restrict progress towards universal health coverage. The objective of the systematic review described in this protocol will be to evaluate and synthesize the evidence concerning the impacts of health systems financing fragmentation in LMICs.

**Methods:**

Literature searches will be conducted in multiple electronic databases, from their inception onwards, including MEDLINE, EMBASE, LILACS, CINAHL, Scopus, ScienceDirect, Scielo, Cochrane Library, EconLit, and JSTOR. Gray literature will be also targeted through searching OpenSIGLE, Google Scholar, and institutional websites (e.g., HMIC, The World Bank, WHO, PAHO, OECD). The search strings will include keywords related to LMICs, health system financing fragmentation, and health system goals. Experimental, quasi-experimental, and observational studies conducted in LMICs and examining health financing fragmentation across any relevant metric (e.g., the presence of different health funders/insurers, risk pooling mechanisms, eligibility categories, benefits packages, premiums) will be included. Studies will be eligible if they compare financing fragmentation in alternative settings or at least two-time points. The primary outcomes will be health system-related goals such as health outcomes (e.g., mortality, morbidity, patient-reported outcome measures) and indicators of access, services utilization, equity, and financial risk protection. Additional outcomes will include intermediate health system objectives (e.g., indicators of efficiency and quality). Two reviewers will independently screen all citations, abstract data, and full-text articles. Potential conflicts will be resolved through discussion and, when necessary, resolved by a third reviewer. The methodological quality (or risk of bias) of selected studies will be appraised using established checklists. Data extraction categories will include the studies’ objective and design, the fragmentation measurement and domains, and health outcomes linked to the fragmentation. A narrative synthesis will be used to describe the results and characteristics of all included studies and to explore relationships and findings both within and between the studies.

**Discussion:**

Evidence on the impacts of health system fragmentation in LMICs is key for identifying evidence gaps and priority areas for intervention. This knowledge will be valuable to health system policymakers aiming to strengthen health systems in LMICs.

**Systematic review registration:**

PROSPERO CRD42020201467

**Supplementary Information:**

The online version contains supplementary material available at 10.1186/s13643-021-01714-5.

## Background

Substantial inequalities in access to quality health services and health outcomes persist between and within low- and middle-income countries (LMICs). Health system fragmentation is a barrier in advancing universal health coverage (UHC) in LMICs and addressing these inequalities. Health system fragmentation can be defined as the “division without explicit means of coordination” of functions (e.g., financing, provision) or agents (e.g., payers, providers) in a health system or its sub-system [1]. It can be further characterized by the existence of many non-integrated entities that operate without synergy and often in competition [1] and encompass a lack of coordination among organizations, functions, and governance systems [2–4].

Fragmentation of health systems financing can be considered across six dimensions (Table 1) [1]. Specifically, these cover the number of different organizations, risk pooling mechanisms, groups of beneficiaries, benefits packages, combinations of premiums, and payment mechanisms in a health system, with increasing numbers considered indicative of higher system fragmentation. Fragmentation may undermine progress towards UHC as health system quality and efficiency can be compromised through multiple providers, diffuse governance arrangements, poor budgetary planning, misalignment of incentives, and duplication and mistargeting of services [5–8]. It may also lead to imbalances in human resources distribution, medical errors associated with fragmented information flows, difficulty standardizing healthcare quality, increased administrative costs, and reduced bargaining power for purchasing [1]. Fragmentation can also contribute to health inequalities when different populations use different systems or financing arrangements with disparate levels of healthcare accessibility and quality [9–14].

**Table 1:** Dimensions of health systems financing fragmentation

Dimension	Description
Organizations	Different organizations offering health financing coverage or insurance to a significant portion of the population
Risk pooling	Different mechanisms that pool or share health financing across population sub-groups and/or across financing organizations
Eligibility	Different eligibility categories for beneficiaries
Benefits	Different benefits packages offered by these organizations
Premiums	Different contributions or premium levels offered by these organizations
Payments	Different payers and payment mechanisms for major provider types

Source: Based on Bossert et al. [1]

Despite the prevalence of health system fragmentation in LMICs and the growing understanding that countries ought to reduce fragmentation to achieve UHC [15], the available literature on the theme seems to be predominantly descriptive and to address the financing fragmentation domains separately, such as a proposed classification for pooling arrangements in health financing systems [16] or a proposed framework on core elements of setting a health benefits package [17]. Many previous reviews have synthesized aspects of the integration or coordination of the healthcare delivery [18–35] or the patient outcomes associated with the fragmentation in the provision of specific health services [36–38], while health financing aspects and whole system approaches are not often addressed. Considering non-overlapping previous contributions and the relevance of the topic, the objective of the systematic review described in this protocol will be to evaluate and synthesize the evidence concerning the impacts of health systems financing fragmentation in LMICs. It will specifically identify what measures and domains of financing fragmentation have been examined in the literature and investigate the impacts of financing fragmentation on health system goals [11].

## Methods/design

The present review protocol is being reported in accordance with the guidelines of the Preferred Reporting Items for Systematic Reviews and Meta-Analyses Protocols (PRISMA-P) statement [39] (see PRISMA-P checklist in Additional file 1). This protocol has been registered in the international prospective register of systematic reviews (PROSPERO) under the registration number CRD42020201467 [40].

### Eligibility criteria

The population, intervention, comparison, outcome, and study design (PICOS) framework is used to structure inclusion and exclusion criteria (Table 2). Eligible populations are any LMIC populations (see Additional file 2), with no age or demographic restrictions. Units of analysis may include patients, providers, insurers, geographical areas, health systems, or sub-systems. Eligible interventions are any relevant measure of health financing fragmentation, which can be generally defined as “the division, without explicit means of coordination, of various dimensions of the health financing and payment control knobs in a given country” [1]. The concept refers to the existence of several non-coordinated funding mechanisms and funding pools within a health system, thus limiting its redistributive capacity through income and risk cross-subsidies (e.g., many small insurance schemes operating under distinct funding pools, covering different populations, charging different premiums, and offering distinct benefits packages) [41, 42].

**Table 2:** Inclusion and exclusion criteria according to PICOS guidelines

Population	Inclusion: Studies conducted in LMICs. The list of eligible countries is provided in Additional file 2, following the World Bank classification of countries by income groups. Exclusion: Studies conducted in high-income countries.
Intervention	Inclusion: Relevant measures of health financing fragmentation (e.g., risk pools, benefits packages, premiums, payment mechanisms to providers, and funding organizations in the health system). Exclusion: Measures not related to health systems fragmentation or concerning fragmentation in healthcare provision (e.g., fragmentation of care index, a metric of dispersion of medical visits across different providers).
Comparison	Inclusion: Any relevant measure of financing fragmentation in alternative settings (i.e., intervention and control groups) or at alternative time points (e.g., before and after an intervention implementation). Exclusion: Studies with no comparator and a single data point
Outcomes	Inclusion: The primary outcomes will be health system-related goals such as health outcomes (e.g., mortality, morbidity, patient-reported outcome measures) and indicators of access, services utilization, equity, and financial risk protection. Additional outcomes will include intermediate health system objectives (e.g., indicators of efficiency and quality). Exclusion: Outcomes not related to health systems’ goals.
Study	Inclusion: Eligible designs are experimental (e.g., randomized controlled trials), quasi-experimental (e.g., interrupted time series, pretest-posttest, regression discontinuity, difference-in-difference, and propensity score matching analyses), or observational studies (e.g., cross-sectional studies, case-control, cohort studies). Included studies will be restricted to articles and working papers published in English, Portuguese, or Spanish and with free access to the full text. There are no date restrictions. Exclusion: Non-empirical, qualitative, or descriptive studies (e.g., editorials, experts’ opinions, meeting reports, news items, case series, case reports, case studies, interview-based studies).

Based on the provided definition, indicators of financing fragmentation can include, but are not limited to, the presence, within the same health system or sub-system, of multiple organizations offering health insurance; multiple risk pool mechanisms for sharing health financing across population sub-groups; multiple eligibility categories for beneficiaries; multiple benefits packages offered to beneficiaries; multiple contributions or premium levels; and/or multiple payers and payment mechanisms for providers [1]. Studies with eligible comparators include those that measure fragmentation in alternative settings (e.g., countries, providers, or population groups) or at alternative time points (e.g., before and after changes) allowing quantification of differences in fragmentation and outcomes. Considering a health systems perspective, the primary outcomes for which data will be sought will be the health system-related goals of improved health outcomes and indicators of access, services utilization, equity, financial risk protection, and responsiveness. Additional outcomes will be the intermediate and instrumental objectives to the broad health systems’ goals, encompassing improved healthcare quality and efficiency in the delivery and organization of health services and the health system administration [43, 44]. The interpretation of outcome categories is provided in the following.

#### Primary outcomes

- *Health outcomes*. Attributes that describe the consequences of disease or a change in an individual’s health status (e.g., mortality, morbidity, patient-reported outcome measures)
- *Financial risk protection*. Measures related to financial hardship from paying for health services (e.g., out-of-pocket health spending, catastrophic health expenditure)
- *Access*. Measures related to the availability and distribution of health services and healthcare resources for the population (e.g., the geographical density of specific services, percentage of births attended by skilled personnel)
- *Services utilization*. Measures such as the number of hospitalizations, medical visits, procedures, or exams performed
- *Equity*. Measures covering inequalities in healthcare access, treatment, or health outcomes across population groups, which can be defined socially, economically, demographically, or geographically
- *Responsiveness*. Measures related to the patients’ satisfaction and expectations towards the health system and health services

#### Additional outcomes

- *Quality*. Measures related to evidence-based practices and processes and patient safety indicators (e.g., hospital-acquired infections, medical error, preventable adverse event)
- *Efficiency*. Measures relating human, physical, or financial inputs employed with the outputs generated (e.g., health expenditure per capita, bed occupancy rate, the average length of stay, comparisons of costs, and outcomes for predefined episodes of care)

### Information sources and search strategy

As a primary source of information, a literature search will be performed on multiple electronic databases (from their inception onwards): MEDLINE (Pubmed), EMBASE, Scopus, LILACS, CINAHL, ScienceDirect, SciELO, Cochrane Library, EconLit, and JSTOR. A secondary source of potentially relevant material will be a search on online libraries of relevant institutions (e.g., HMIC, The World Bank, WHO, PAHO, and OECD) and of gray or difficult to locate literature, such as OpenSIGLE and Google Scholar. Hand-search on the reference lists of included studies, relevant reviews, and policy documents will also be performed, and content experts and authors who are prolific in the field will be contacted. A comprehensive search strategy will be adopted, including a broad range of terms and keywords across four domains: (i) “fragmentation” and related phrases (e.g., “segmentation” and “non-coordination”), (ii) health system settings (e.g., “health” or “healthcare”), (iii) health financing aspects terms (e.g., “revenue collection”, “eligibility criteria”, “benefits packages”, “insurance coverage”, “risk pools”, “payment mechanisms”, “spending on health”), and (iv) terms related to LMICs, and the name of eligible countries. A draft search strategy for MEDLINE (Pubmed) is detailed in Additional file 3.

### Screening and selection process

Title screening will be undertaken separately by two researchers using the Mendeley Software. All potentially relevant studies and records lacking information to determine eligibility (e.g., abstract not available) will be retained for further assessment. Abstract screening will be undertaken independently by two reviewers (MM and MC). Disagreements will be discussed and if no consensus is reached discrepancies will be resolved by a third reviewer (TH). One reviewer will undertake the full-text assessment (MM), and a second reviewer will check the assessment (MC). Disagreements will be discussed and resolved with a third reviewer (TH). A PRISMA flow chart showing details of studies included and excluded at each stage of the study selection process will be provided [45].

### Data extraction

Data of selected studies will be extracted by one researcher (MM) and verified by a second researcher (MC). Data will be extracted into a spreadsheet using a designed data collection form. Exact data extraction will be further refined based on the types of studies identified, but key information to be extracted will include the following:

- Basic information (authors, year of publication, journal of publication, country)
- Administrative information (funding sources, ethical approval, conflicts of interest)
- Study objective and research question(s)
- Theoretical framework (when available)
- Health service or health system characteristics
- Study participants (e.g., number and characteristics) and sampling strategy
- Data sources
- Fragmentation definition (when available) and measured fragmentation domains
- Study design and analytical strategy
- Comparators
- Outcome measures and reported effect sizes
- Overall findings
- Key study assumptions and limitations
- Information related to the risk of bias domains and study quality (see the following)

### Quality assessment

The risk of bias and quality of selected studies will be assessed using an appropriate tool selected following a review of included study designs. This may include an EPOC tool for applicable study designs (i.e., randomized trials, non-randomized trials, controlled before-after, and interrupted time-series studies) [46], or other published approaches such as ROBINS-I [47] or Newcastle-Ottawa Scale (NOS) [48]. One reviewer will conduct the quality assessment (MM) and a second researcher will review and highlight any discrepancies (MC). Disagreements will be resolved with a third reviewer (TH). Based on the risk of bias domains applicable to the selected designs, studies will be classified as having a low, high, or unclear risk of bias. The assessment of the overall strength of evidence will be assessed by the research team. Quality assessment will also take into account that a higher number of papers in an area that does not necessarily indicate greater strength of evidence, only where more work has been carried out.

### Data analysis/synthesis

A meta-analysis will be carried out if included studies are sufficiently homogeneous. Risk ratio (RR) or odds ratio (OR) will be used to determine the intervention’s effect on dichotomous outcomes and mean differences will be used for continuous outcomes, considering a 95% confidence interval (CI). If feasible and appropriate, outcome data will be used to perform random-effects meta-analyses because heterogeneity is expected a priori. The random-effects model assumes the study-level effect estimates follow a normal distribution, considering both within-study and between-study variation.

If a substantial diversity of populations, research designs, comparators, and outcomes measures is observed among selected studies, precluding a quantitative synthesis, a systematic narrative synthesis will be employed to summarize and explain the characteristics and findings of selected studies. The Synthesis Without Meta-analysis (SWiM) guideline will be consulted to ensure transparent reporting of interventions through narrative synthesis [49]. A description and rationale will be provided for grouping studies for synthesis (e.g., according to study design, risk of bias level, intervention context, outcome types), aiming to minimize their methodological or conceptual diversity. Established metrics will be used to measure the direction and magnitude of interventions’ effect (e.g., RR, OR, risk differences, mean differences). Summary tables, figures, and structured narratives will be used to descriptively summarize and compare each included study and examine the heterogeneity across studies in a clear format. Taking into account the systematic review objective, to evaluate and synthesize the evidence concerning the impacts of health systems financing fragmentation in LMICs, we anticipate that tables will include key information regarding the interventions setting, the studies methodological characterization (e.g., study design and method, participants in the intervention, and control groups), and the domains of fragmentation addressed (e.g., risk pooling, benefits packages, premiums, eligibility categories).

To assess the presence of reporting bias, we will examine whether the selected studies have a Clinical Trial Register at the International Clinical Trials Registry Platform (ICTRP) of the World Health Organization (https://www.who.int/clinical-trials-registry-platform/unambiguous-trial-identification), a pre-registered protocol or statistical analysis plan published before the recruitment of participants into the study. The correspondence between planned and reported outcome measures, analyses, and sub-groups of participants will be examined.

Given that systematic review protocols are usually iterative documents and adjustments during the review process are possible, all relevant amendments to this protocol will be documented in detail, including the date, description, and rationale of each change.

### Confidence in cumulative evidence

The Grading of Recommendations Assessment, Development, and Evaluation (GRADE) system will be used to seek a transparent and structured approach for summarizing and rating the quality of evidence for each outcome presented in the systematic review [50]. Experimental designs start as high-quality evidence and observational designs as low-quality evidence in terms of supporting estimates of intervention effects, with additional factors possibly leading to the rating up or down the quality of evidence (e.g., risk of bias, consistency, directness, precision, publication bias, plausible confounding, large effect). The overall quality of evidence will be rated as high, moderate, low, or very low for a given outcome, according to the level of certainty and the likely impact of further research on the confidence of estimates of effect [51].

## Discussion

This protocol represents an important starting point towards performing a systematic review to examine and synthesize the effects of financing fragmentation on health system goals in LMICs. Publishing the protocol before carrying out the systematic review allows a peer-review evaluation generally leading to quality improvements on the planned review [52]. The relevance of investigating the topic arises because, despite descriptive evidence suggesting that health financing is an important determinant of health system fragmentation and that fragmentation remains a challenge for LMICs in addressing inequalities, improving efficiency, and advancing in UHC, estimates of the impact of fragmentation on health system goals remain unclear [1–4, 15, 16].

There are likely to be limitations at the study level that may lead to reduced internal and external validity in selected studies, such as the potential for confounding of the effect of the intervention, the risk of bias in the selection of participants into the study, and in the definition and classification of intervention and control groups, deviations from intended interventions and important co-interventions not balanced across comparison groups, bias due to missing data and to the outcome measurement, and selective reporting. In this regard, an appropriate tool will be applied to assess and report the limitations at the study level in a clear and standard format, according to the designs of selected studies [46–48].

A potential drawback is the expected complexity of interventions tackling health system financing fragmentation, whereby diffuse or weak effects could be attributable to different policy designs regarding funding mechanisms, target population, benefits package offered, enrollment strategies, etc. Health system interventions not rarely cover both demand-side and supply-side components, and involve multiple stakeholders, and its impacts depend on users’ behaviors, in addition to the characteristics of the health system and the social, economic, and political context where the health system it is inserted. Objectives sought by health financing interventions may also not be fulfilled due to the influence of several market aspects, such as distribution and level of competition of health providers, the users’ demand by types of services, and the existence of regulation and information for users, among others. That is, besides being difficult to measure isolated effects of complex and multi-element interventions, the implementation of a similar intervention can vary from place to place, as well as the time for it to be truly integrated into a health system [41, 53, 54].

There are also key limitations to the systematic review. The expected diversity of participants, designs, and outcomes pose challenges in synthesizing and generalizing findings. It is likely that many studies will be of low quality and suffer from methodological weaknesses given the LMIC focus and the research topic. A wide approach to searching for literature, including 17 electronic databases, will maximize the likelihood of capturing relevant studies, but there is the potential that relevant studies will be missed. Research biases may also occur, but these will be minimized by using two authors for screening, selection, quality assessment, and analysis of studies, and employing established tools for quality assessment.

The systematic review presented in this protocol will present the evidence base regarding the impacts of financing fragmentation in LMICs in a transparent and structured format, allowing identification of potential research gaps and methodological shortcomings. For researchers, the synthesis will provide an overview of the current state of knowledge in the topic, highlight methodological weaknesses of existing studies, and indicate where further research is needed. The findings might therefore help to steer further research and lay the groundwork for future empirical studies on the topic. For policymakers, the systematic review intends to outline areas for intervention and, by discussing potential pathways by which fragmentation impacts health system goals, to inform efforts to strengthen health systems and advance progress towards UHC.

## Supplementary Information

**Additional file 1.** PRISMA-P Checklist.

**Additional file 2.** List of eligible Low-and-Middle Income Countries.

**Additional file 3.** Strategy adopted for the search on PubMed.

## Data Availability

All data generated or analyzed during this study will be available in another published article.

## Abbreviations

LMICs: Low-and middle-income countries
HMIC: Healthcare Management Information Consortium
OECD: Organisation for Economic Co-operation and Development
PICOS: Population, intervention, comparison, outcome, and study design
PRISMA: Preferred Reporting Items for Systematic Review and Meta-Analysis
PRISMA-P: Preferred Reporting Items for Systematic Review and Meta-Analysis Protocols
UHC: Universal health coverage
WHO: World Health Organization

## References

[1] Bossert T, Blanchet N, Sheetz S, Pinto D, Cali J, Cuevas RP (2014). Comparative review of health system integration in selected countries in Latin America. Technical Note no. 585.

[2] Lewis RQ (2010). Nuffield Trust for Research and Policy Studies in Health Services, King Edward’s Hospital Fund for London. Where next for integrated care organisations in the English NHS?.

[3] Wallace C. An exploration of health and social care service integration in a deprived South Wales Area [Thesis] Coventry, Eng.: Coventry University in collaboration with the University of Worcester; 2009. https://core.ac.uk/reader/1914778. Accessed 7 Jul 2020.

[4] Contandriopoulos A-P, santé U de MG de recherche interdisciplinaire en. The integration of health care: dimensions and implementation: working paper. GRIS, Université de Montréal; 2004. 27 p.

[5] Greer SL, Méndez CA (2015). Universal health coverage: a political struggle and governance challenge. Am J Public Health..

[6] Greer S, Wismar M, Figueras J (2016). Strengthening health system governance: better policies, stronger performance.

[7] Travis P, Egger D, Davies P, Mechbal A (2002). Towards better stewardship: concepts and critical issues.

[8] Stange KC (2009). The problem of fragmentation and the need for integrative solutions. Ann Fam Med.

[9] Collins C, Green A (2014). Valuing health systems: a framework for low- and middle-income countries.

[10] WHO. The World Health Report 2000 - health systems: improving performance. Geneva, Switzerland: World Health Organization, 2000. https://www.who.int/whr/2000/en/. Accessed 15 Jul 2020.

[11] WHO. Everybody’s business: strengthening health systems to improve health outcomes. Geneva, Switzerland: World Health Organization, 2007. https://apps.who.int/iris/handle/10665/43918. Accessed 15 Jul 2020.

[12] WHO. The World Health Report 2008 - primary health care (now more than ever). Geneva, Switzerland: World Health Organization, 2008. https://www.who.int/whr/2008/en/. Accessed 18 Jul 2020.

[13] WHO. Tracking universal health coverage: first global monitoring report. Geneva, Switzerland: World Health Organization, 2015. http://www.who.int/health_financing/documents/tracking-uhc/en/. Accessed 18 Jul 2020.

[14] WHO. WHO Framework on integrated people-centred health services. Geneva, Switzerland: World Health Organization, 2016. http://www.who.int/servicedeliverysafety/areas/people-centred-care/en/. Accessed 19 Jul 2020.

[15] Mladovsky P (2020). Fragmentation by design: universal health coverage policies as governmentality in Senegal. Soc Sci Med.

[16] Mathauer I, Saksena P, Kutzin J (2019). Pooling arrangements in health financing systems: a proposed classification. Int J Equity Health..

[17] Glassman A, Giedion U, Sakuma Y, Smith PC (2016). Defining a health benefits package: what are the necessary processes?. Health Syst Reform.

[18] Bautista MAC, Nurjono M, Lim YW, Dessers E, Vrijhoef HJ (2016). Instruments measuring integrated care: a systematic review of measurement properties. Milbank Q..

[19] Strandberg-Larsen M, Krasnik A. Measurement of integrated healthcare delivery: a systematic review of methods and future research directions. Int J Integr Care. 2009 https://www.ncbi.nlm.nih.gov/pmc/articles/PMC2663702/. Accessed 7 Jul 2020;9(1). 10.5334/ijic.305. 10.5334/ijic.305 PMC2663702 19340325

[20] Peterson K, Anderson J, Bourne D, Charns MP, Gorin SS, Hynes DM, McDonald KM, Singer SJ, Yano EM (2019). Health care coordination theoretical frameworks: a systematic scoping review to increase their understanding and use in practice. J Gen Intern Med..

[21] Baxter S, Johnson M, Chambers D, Sutton A, Goyder E, Booth A (2018). The effects of integrated care: a systematic review of UK and international evidence. BMC Health Serv Res..

[22] Lewis ME, Myhra LL (2017). Integrated care with indigenous populations: a systematic review of the literature. Am Indian Alsk Native Ment Health Res..

[23] Shahidullah JD, Carlson JS, Haggerty D, Lancaster BM (2018). Integrated care models for ADHD in children and adolescents: a systematic review. Fam Syst Health..

[24] Shahzad M, Upshur R, Donnelly P, Bharmal A, Wei X, Feng P, Brown AD (2019). A population-based approach to integrated healthcare delivery: a scoping review of clinical care and public health collaboration. BMC Public Health..

[25] dos Santos-Melo GZ, de Andrade SR, Ruoff AB (2018). A integração de saúde entre fronteiras internacionais: uma revisão integrativa. Acta Paul Enferm..

[26] Dudley L, Garner P (2011). Strategies for integrating primary health services in low- and middle-income countries at the point of delivery. Cochrane Database Syst Rev..

[27] Rygh EM, Hjortdahl P (2007). Continuous and integrated health care services in rural areas. A literature study. Rural Remote Health.

[28] Gregg A, Tutek J, Leatherwood MD, Crawford W, Friend R, Crowther M, McKinney R (2019). Systematic review of community paramedicine and EMS mobile integrated health care interventions in the United States. Popul Health Manag..

[29] Aubin M, Giguère A, Martin M, Verreault R, Fitch MI, Kazanjian A, Carmichael PH, Cochrane Effective Practice and Organisation of Care Group (2012). Interventions to improve continuity of care in the follow-up of patients with cancer. Cochrane Database Syst Rev..

[30] McKibbon KA, Lokker C, Handler SM, Dolovich LR, Holbrook AM, O’Reilly D (2012). The effectiveness of integrated health information technologies across the phases of medication management: a systematic review of randomized controlled trials. J Am Med Inform Assoc..

[31] Mackie S, Darvill A (2016). Factors enabling implementation of integrated health and social care: a systematic review. Br J Community Nurs..

[32] Wakida EK, Talib ZM, Akena D, Okello ES, Kinengyere A, Mindra A (2018). Barriers and facilitators to the integration of mental health services into primary health care: a systematic review. Syst Rev.

[33] McKillop A, Shaw J, Sheridan N, Gray CS, Carswell P, Wodchis WP, Connolly M, Denis JL, Baker GR, Kenealy T (2017). Understanding the attributes of implementation frameworks to guide the implementation of a model of community-based integrated health care for older adults with complex chronic conditions: a metanarrative review. Int J Integr Care..

[34] Reynolds HW, Sutherland EG (2013). A systematic approach to the planning, implementation, monitoring, and evaluation of integrated health services. BMC Health Serv Res..

[35] Black DR (2017). Preparing the workforce for integrated healthcare: a systematic review. Soc Work Health Care..

[36] Juo Y-Y, Sanaiha Y, Khrucharoen U, Chang BH, Dutson E, Benharash P (2019). Care fragmentation is associated with increased short-term mortality during postoperative readmissions: a systematic review and meta-analysis. Surgery..

[37] Snow K, Galaviz K, Turbow S (2020). Patient outcomes following interhospital care fragmentation: a systematic review. J Gen Intern Med..

[38] Wang V, Diamantidis CJ, Wylie J, Greer RC (2017). Minding the gap and overlap: a literature review of fragmentation of primary care for chronic dialysis patients. BMC Nephrol..

[39] Shamseer L, Moher D, Clarke M, Ghersi D, Liberati A, Petticrew M, Shekelle P, Stewart LA, the PRISMA-P Group (2015). Preferred reporting items for systematic review and meta-analysis protocols (PRISMA-P) 2015: elaboration and explanation. BMJ..

[40] Jud L, Fotouhi J, Andronic O, Aichmair A, Osgood G, Navab N, Farshad M (2020). Applicability of augmented reality in orthopedic surgery - a systematic review. BMC Musculoskelet Disord..

[41] Kutzin J (2001). A descriptive framework for country-level analysis of health care financing arrangements. Health Policy (Amsterdam, Netherlands).

[42] McIntyre D, Garshong B, Mtei G, Meheus F, Thiede M, Akazili J, Ally M, Aikins M, Mulligan J-A, Goudge J (2008). Beyond fragmentation and towards universal coverage: insights from Ghana, South Africa and the United Republic of Tanzania. Bull World Health Organ.

[43] Kutzin J (2013). Health financing for universal coverage and health system performance: concepts and implications for policy. Bull World Health Organ..

[44] WHO. Monitoring and evaluation of health systems strengthening: an operational framework. Geneva, Switzerland: World Health Organization [Internet]. 2009 [cited 2021 Apr 6]. Available from: https://www.who.int/healthinfo/HSS_MandE_framework_Nov_2009.pdf

[45] Page MJ, McKenzie JE, Bossuyt PM, Boutron I, Hoffmann TC, Mulrow CD, et al. The PRISMA 2020 statement: an updated guideline for reporting systematic reviews. BMJ. 2021:n71. 10.1136/bmj.n71. 10.1136/bmj.n71 PMC8005924 33782057

[46] Cochrane Effective Practice and Organisation of Care (EPOC). [Suggested risk of bias criteria for EPOC reviews]. EPOC Resources for review authors, 2017. https://epoc.cochrane.org/resources/epoc-resources-review-authors. Accessed 20 June 2020.

[47] Sterne JA, Hernán MA, Reeves BC, Savović J, Berkman ND, Viswanathan M, et al. ROBINS-I: a tool for assessing risk of bias in non-randomised studies of interventions. BMJ. 2016:355. 10.1136/bmj.i4919. 10.1136/bmj.i4919 PMC5062054 27733354

[48] Stang A (2010). Critical evaluation of the Newcastle-Ottawa scale for the assessment of the quality of nonrandomized studies in meta-analyses. Eur J Epidemiol.

[49] Campbell M, McKenzie JE, Sowden A, Katikireddi SV, Brennan SE, Ellis S, Hartmann-Boyce J, Ryan R, Shepperd S, Thomas J, Welch V, Thomson H (2020). Synthesis without meta-analysis (SWiM) in systematic reviews: reporting guideline. BMJ.

[50] Guyatt GH, Oxman AD, Vist GE, Kunz R, Falck-Ytter Y, Alonso-Coello P, Schünemann HJ (2008). GRADE: an emerging consensus on rating quality of evidence and strength of recommendations. BMJ.

[51] Guyatt G, Oxman AD, Akl EA, Kunz R, Vist G, Brozek J, Norris S, Falck-Ytter Y, Glasziou P, deBeer H, Jaeschke R, Rind D, Meerpohl J, Dahm P, Schünemann HJ (2011). GRADE guidelines: 1. Introduction—GRADE evidence profiles and summary of findings tables. J Clin Epidemiol.

[52] Allers K, Hoffmann F, Mathes T, Pieper D (2018). Systematic reviews with published protocols compared to those without: more effort, older search. J Clin Epidemiol.

[53] Atun R, de Jongh T, Secci F, Ohiri K, Adeyi O (2010). Integration of targeted health interventions into health systems: a conceptual framework for analysis. Health Policy Plann.

[54] Walt G, Gilson L (1994). Reforming the health sector in developing countries: the central role of policy analysis. Health Policy Plann.

