# Supplementary material for: The impacts of health systems financing fragmentation in low- and middle-income countries: a systematic review protocol
Source: Syst Rev. 2021 Jun 2;10:164. doi: 10.1186/s13643-021-01714-5 (PMC8170990; doi:10.1186/s13643-021-01714-5)
Supplement: Supplementary file 1 — Additional file 1. PRISMA-P Checklist. [file 13643_2021_1714_MOESM1_ESM.docx]

**Additional File 1** PRISMA-P Checklist

# **This checklist has been adapted for use with protocol submissions to *Systematic Reviews* from Table 3 in Moher D et al**:**** Preferred reporting items for systematic review and meta-analysis protocols (PRISMA-P) 2015 statement. *Systematic Reviews* 2015 ****4****:1

| **Section/topic** | **#** | **Checklist item** | **Information reported** | | **Line number(s)** |
| --- | --- | --- | --- | --- | --- |
|  |  |  | **Yes** | **No** |  |
|  | | | | | |
|  | | | | | |
| Identification | 1a | Identify the report as a protocol of a systematic review | X |  | p. 1, line 1 |
| Update | 1b | If the protocol is for an update of a previous systematic review, identify as such |  |  | NA |
| **Registration** | 2 | If registered, provide the name of the registry (e.g., PROSPERO) and registration number in the Abstract | X |  | p. 2, line 17 |
|  | | | | | |
| Contact | 3a | Provide name, institutional affiliation, and e-mail address of all protocol authors; provide physical mailing address of corresponding author | X |  | p. 16, lines 18-21 |
| Contributions | 3b | Describe contributions of protocol authors and identify the guarantor of the review | X |  | p. 16, lines 11-13 |
| **Amendments** | 4 | If the protocol represents an amendment of a previously completed or published protocol, identify as such and list changes; otherwise, state plan for documenting important protocol amendments |  |  | NA |
|  | | | | | |
| Sources | 5a | Indicate sources of financial or other support for the review | X |  | p. 16, lines 7-9 |
| Sponsor | 5b | Provide name for the review funder and/or sponsor | X |  | p. 16, lines 7-9 |
| Role of sponsor/funder | 5c | Describe roles of funder(s), sponsor(s), and/or institution(s), if any, in developing the protocol |  |  | NA |
|  | | | | | |
| **Rationale** | 6 | Describe the rationale for the review in the context of what is already known | X |  | p. 3 lines 18-23; p. 4, lines 1-6 |
| **Objectives** | 7 | Provide an explicit statement of the question(s) the review will address with reference to participants, interventions, comparators, and outcomes (PICO) | X |  | p. 1, lines 8-9; |
|  | | | | | |
| **Eligibility criteria** | 8 | Specify the study characteristics (e.g., PICO, study design, setting, time frame) and report characteristics (e.g., years considered, language, publication status) to be used as criteria for eligibility for the review | X |  | p. 5, lines 8-23; p. 6-8 |
| **Information sources** | 9 | Describe all intended information sources (e.g., electronic databases, contact with study authors, trial registers, or other grey literature sources) with planned dates of coverage | X |  | p. 8, lines 2-6; p. 9, lines 1-10 |
| **Search strategy** | 10 | Present draft of search strategy to be used for at least one electronic database, including planned limits, such that it could be repeated | X |  | Additional file 3 |
|  | | | | | |
| Data management | 11a | Describe the mechanism(s) that will be used to manage records and data throughout the review | X |  | p. 9, lines 12-21 |
| Selection process | 11b | State the process that will be used for selecting studies (e.g., two independent reviewers) through each phase of the review (i.e., screening, eligibility, and inclusion in meta-analysis) | X |  | p. 9, lines 12-21 |
| Data collection process | 11c | Describe planned method of extracting data from reports (e.g., piloting forms, done independently, in duplicate), any processes for obtaining and confirming data from investigators | X |  | p. 10, lines 1-18 |
| **Data items** | 12 | List and define all variables for which data will be sought (e.g., PICO items, funding sources), any pre-planned data assumptions and simplifications | X |  | p. 6, lines 4-10 |
| **Outcomes and prioritization** | 13 | List and define all outcomes for which data will be sought, including prioritization of main and additional outcomes, with rationale | X |  | p. 6, lines 4-23; p. 7, lines 1-8 |
| **Risk of bias in individual studies** | 14 | Describe anticipated methods for assessing risk of bias of individual studies, including whether this will be done at the outcome or study level, or both; state how this information will be used in data synthesis | X |  | p. 10, lines 21-23; p. 11, lines 1-8; p. 13, lines 1-9 |
|  | | | | | |
| **Synthesis** | 15a | Describe criteria under which study data will be quantitatively synthesized | X |  | p. 11, lines 11-16 |
|  | 15b | If data are appropriate for quantitative synthesis, describe planned summary measures, methods of handling data, and methods of combining data from studies, including any planned exploration of consistency (e.g., *I* ^2^, Kendall’s tau) | X |  | p. 11, lines 11-16 |
|  | 15c | Describe any proposed additional analyses (e.g., sensitivity or subgroup analyses, meta-regression) | X |  | p. 11, lines 21-23 |
|  | 15d | If quantitative synthesis is not appropriate, describe the type of summary planned | X |  | p. 11, lines 17-23; p. 12, lines 1-13 |
| **Meta-bias(es)** | 16 | Specify any planned assessment of meta-bias(es) (e.g., publication bias across studies, selective reporting within studies) | X |  | p. 12, lines 14-19 |
| **Confidence in cumulative evidence** | 17 | Describe how the strength of the body of evidence will be assessed (e.g., GRADE) | X |  | p. 13, lines 1-9 |
