# Supplementary material for: The impacts of health systems financing fragmentation in low- and middle-income countries: a systematic review protocol
Source: Syst Rev. 2021 Jun 2;10:164. doi: 10.1186/s13643-021-01714-5 (PMC8170990; doi:10.1186/s13643-021-01714-5)
Supplement: Supplementary file 2 — Additional file 2. List of eligible Low-and-Middle Income Countries. [file 13643_2021_1714_MOESM2_ESM.docx]

**Additional File 2** List of eligible Low-and-Middle Income Countries

| 1. Afghanistan 2. Albania 3. Algeria 4. American Samoa 5. Angola 6. Antigua and Barbuda 7. Argentina 8. Armenia 9. Azerbaijan 10. Bangladesh 11. Barbados 12. Belarus 13. Belize 14. Benin 15. Bhutan 16. Bolivia 17. Bosnia and Herzegovina 18. Botswana 19. Brazil 20. Bulgaria 21. Burkina Faso 22. Burundi 23. Cambodia 24. Cameroon 25. Cape Verde 26. Central African Republic 27. Chad 28. Chile 29. China 30. Colombia 31. Comoros 32. Congo 33. Costa Rica 34. Cote d'Ivoire 35. Croatia 36. Cuba 37. Czech republic 38. Democratic Republic of Congo 39. Djibouti 40. Dominica 41. Dominican Republic 42. Ecuador 43. Egypt 44. El Salvador 45. Equatorial Guinea 46. Eritrea 47. Estonia 48. Ethiopia 49. Fiji 50. Gabon 51. Gambia 52. Georgia 53. Ghana 54. Grenada 55. Guatemala 56. Guinea 57. Guinea-Bissau 58. Guyana 59. Haiti 60. Honduras 61. Hungary 62. ierra Leone 63. India 64. Indonesia 65. Iran 66. Iraq 67. Jamaica 68. Jordan 69. Kazakhstan 70. Kenya 71. Kiribati 72. Kyrgyzstan 73. Lao 74. Latvia 75. Lebanon 76. Lesotho 77. Liberia 78. Libya 79. Lithuania 80. Macedonia 81. Madagascar 82. Malawi 83. Malaysia 84. Maldives 85. Mali 86. Marshall Islands 87. Mauritania 88. Mauritius 89. Mayotte 90. Mexico 91. Micronesia 92. Moldova 93. Mongolia 94. Morocco 95. Mozambique 96. Myanmar 97. Namibia 98. Nepal 99. Nicaragua 100. Niger 101. Nigeria 102. North Korea 103. Northern Mariana Islands 104. Oman 105. Pakistan 106. Palau 107. Panama 108. Papua New Guinea 109. Paraguay 110. Peru 111. Philippines 112. Poland 113. Romania 114. Russia 115. Rwanda 116. Saint Kitts and Nevis 117. Saint Lucia 118. Saint Vincent and the Grenadines 119. Samoa 120. Sao Tome and Principe 121. SenegalS 122. Serbia and Montenegro 123. Seychelles 124. Slovak Republic 125. Solomon Islands 126. Somalia 127. South Africa 128. Sri Lanka 129. SudanTajikistan 130. Suriname 131. Swaziland 132. Syria 133. Tanzania 134. Thailand 135. Timor-Leste 136. Togo 137. Tonga 138. Trinidad and Tobago 139. Tunisia 140. Turkey 141. Turkmenistan 142. Uganda 143. Ukraine 144. Uruguay 145. Uzbekistan 146. Vanuatu 147. Venezuela 148. Vietnam 149. West Bank and Gaza 150. Yemen 151. Yugoslav 152. Zaire 153. Zambia 154. Zimbabwe |
| --- |
