# Supplementary material for: The impacts of health systems financing fragmentation in low- and middle-income countries: a systematic review protocol
Source: Syst Rev. 2021 Jun 2;10:164. doi: 10.1186/s13643-021-01714-5 (PMC8170990; doi:10.1186/s13643-021-01714-5)
Supplement: Supplementary file 3 — Additional file 3. Strategy adopted for the search on PubMed. [file 13643_2021_1714_MOESM3_ESM.docx]

**Additional File 3** Strategy adopted for the search on PubMed.

| The search was performed on June 11, 2020. Limits to English, Portuguese, and Spanish language were applied. No limits were applied on the date of publication, article type or text availability. The search string used in PubMed’s query box is presented below:  (((“fragmentation”[Title/Abstract] OR “fragmented”[Title/Abstract] OR “segmentation”[Title/Abstract] OR “segmented”[Title/Abstract] OR “non-coordinated”[Title/Abstract] OR “non-coordination”[Title/Abstract] OR “uncoordinated”[Title/Abstract] OR “incoordination”[Title/Abstract] OR “non-integrated”[Title/Abstract] OR “non-integration”[Title/Abstract] OR “detached”[Title/Abstract] OR “disjointed”[Title/Abstract] OR “disconnected”[Title/Abstract] OR “unconnected”[Title/Abstract] OR “incoherent”[Title/Abstract] OR "intermittent"[Title/Abstract] OR "sporadic"[Title/Abstract] OR "irregular"[Title/Abstract] OR "fragmentary"[Title/Abstract] OR "discontinu*"[Title/Abstract] OR "isolated"[Title/Abstract] OR "patchy"[Title/Abstract] OR "scattered"[Title/Abstract] OR "occasional"[Title/Abstract] OR "episodic"[Title/Abstract] OR “duplic*”[Title/Abstract] OR “redundant”[Title/Abstract] OR “redundancy”[Title/Abstract] OR “diffuse”[Title/Abstract] OR “discontinu*”[Title/Abstract] OR “interrupt*”[Title/Abstract] OR “transitioning”[Title/Abstract] OR “referral system*”[Title/Abstract] OR “counter-referral system*”[Title/Abstract] OR “patient handover*”[Title/Abstract] OR “patient hand-over*”[Title/Abstract] OR “clinical handover*”[Title/Abstract] OR “public-private”[Title/Abstract] OR “public/private”[Title/Abstract] OR “public and private”[Title/Abstract] OR “decentralized”[Title/Abstract] OR “deconcentrated”[Title/Abstract] OR “deconcentration”[Title/Abstract] OR “decentralised”[Title/Abstract] OR “decentralization”[Title/Abstract] OR “decentralisation”[Title/Abstract] OR “concession contract*”[Title/Abstract] OR “concession agreement*”[Title/Abstract] OR “integrat*”[Title/Abstract] OR “coordinat*”[Title/Abstract] OR “co-ordinat*”[Title/Abstract]) AND (“health*”[Title/Abstract] OR “healthcare”[Title/Abstract])) AND (“revenue collection”[Title/Abstract] OR “tax-based”[Title/Abstract] OR “general tax”[Title/Abstract] OR “general taxation”[Title/Abstract] OR “general taxes”[Title/Abstract] OR “contribution mechanism*”[Title/Abstract] OR “income”[Title/Abstract] OR “fiscal waiver”[Title/Abstract] OR “tax spending”[Title/Abstract] OR “taxes”[Title/Abstract] OR “fee waiver*”[Title/Abstract] OR “subsid*”[Title/Abstract] OR “non-contributory transfer*”[Title/Abstract] OR “health care reform”[Title/Abstract] OR “purchasing”[Title/Abstract] OR “health procurement”[Title/Abstract] OR “health care procurement”[Title/Abstract] OR “bargaining power”[Title/Abstract] OR “eligib*”[Title/Abstract] OR “qualified”[Title/Abstract] OR “qualify”[Title/Abstract] OR “allowed”[Title/Abstract] OR “entitled”[Title/Abstract] OR “authorised”[Title/Abstract] OR “authorized”[Title/Abstract] OR “approved”[Title/Abstract] OR “permitted”[Title/Abstract] OR “benefit package*”[Title/Abstract] OR “benefits package*”[Title/Abstract] OR “insurance benefit*”[Title/Abstract] OR “beneficiar*”[Title/Abstract] OR “service user*”[Title/Abstract] OR “health basket”[Title/Abstract] OR “benefit basket”[Title/Abstract] OR “service basket”[Title/Abstract] OR “health care package*”[Title/Abstract] OR “healthcare package”[Title/Abstract] OR “essential health services”[Title/Abstract] OR “basic health services”[Title/Abstract] OR “package of services”[Title/Abstract] OR “health insurer*”[Title/Abstract] OR “multiple insurer*”[Title/Abstract] OR “health insurance”[Title/Abstract] OR “social insurance”[Title/Abstract] OR “social protection”[Title/Abstract] OR “community-insurance”[Title/Abstract] OR “micro-insurance”[Title/Abstract] OR “co-insurance”[Title/Abstract] OR “private insurance”[Title/Abstract] OR “private health”[Title/Abstract] OR “public insurance”[Title/Abstract] OR “public health insurance”[Title/Abstract] OR “public health plan*”[Title/Abstract] OR “public health scheme*”[Title/Abstract] OR “fund pooling”[Title/Abstract] OR “pooled fund*”[Title/Abstract] OR “insurance pool*”[Title/Abstract] OR “insurance pooling”[Title/Abstract] OR “risk pool*”[Title/Abstract] OR “risk pooling”[Title/Abstract] OR “financial protection”[Title/Abstract] OR “risk protection”[Title/Abstract] OR “cost sharing”[Title/Abstract] OR “risk sharing”[Title/Abstract] OR “cost-sharing”[Title/Abstract] OR “risk-sharing”[Title/Abstract] OR “out-of-pocket”[Title/Abstract] OR “OOP”[Title/Abstract] OR “catastrophic spending”[Title/Abstract] OR “catastrophic health spending”[Title/Abstract] OR “catastrophic health expenditure”[Title/Abstract] OR “catastrophic payment”[Title/Abstract] OR “impoverishment”[Title/Abstract] OR “fee-for-service”[Title/Abstract] OR “FFS”[Title/Abstract] OR “pay for performance”[Title/Abstract] OR “pay-for-performance”[Title/Abstract] OR “P4P”[Title/Abstract] OR “value-based payment”[Title/Abstract] OR “bundled payment”[Title/Abstract] OR “bundled payments”[Title/Abstract] OR “capitation”[Title/Abstract] OR “payment mechanism”[Title/Abstract] OR “global budget”[Title/Abstract] OR “global budgeting”[Title/Abstract] OR “case-based payment”[Title/Abstract] OR “prepaid”[Title/Abstract] OR “prepayment”[Title/Abstract] OR “reimbursement mechanism”[Title/Abstract] OR “multi-payer*”[Title/Abstract] OR “multi payer*”[Title/Abstract] OR “multiple payers”[Title/Abstract] OR “premium level*”[Title/Abstract] OR “copayment*”[Title/Abstract] OR “co-payment*”[Title/Abstract] OR “copay”[Title/Abstract] OR “co-pay”[Title/Abstract] OR “deductible*”[Title/Abstract] OR “health care utilization”[Title/Abstract] OR “healthcare utilization”[Title/Abstract] OR “service utilization”[Title/Abstract] OR “health care utilisation”[Title/Abstract] OR “healthcare utilisation”[Title/Abstract] OR “service utilisation”[Title/Abstract] OR “insurance coverage”[Title/Abstract] OR “health coverage”[Title/Abstract] OR “care coverage”[Title/Abstract] OR “service coverage”[Title/Abstract] OR “inpatient cover*”[Title/Abstract] OR “outpatient cover*”[Title/Abstract] OR “dental cover*”[Title/Abstract] OR "compulsory insurance"[Title/Abstract] OR "compulsory health"[Title/Abstract] OR "compulsory affiliation"[Title/Abstract] OR "compulsory scheme*"[Title/Abstract] OR “treatment coverage”[Title/Abstract] OR “qualifying coverage”[Title/Abstract] OR “financing coverage”[Title/Abstract] OR “universal coverage”[Title/Abstract] OR “universal health coverage”[Title/Abstract] OR “UHC”[Title/Abstract] OR “minimum essential coverage”[Title/Abstract] OR “eligible for coverage”[Title/Abstract] OR “social protection”[Title/Abstract] OR “social assistance”[Title/Abstract] OR “welfare”[Title/Abstract] OR “means test”[Title/Abstract] OR “the poor”[Title/Abstract] OR “the vulnerable”[Title/Abstract] OR “health system financing”[Title/Abstract] OR “health systems financing*”[Title/Abstract] OR “service financing”[Title/Abstract] OR “services financing”[Title/Abstract] OR “health financing”[Title/Abstract] OR “healthcare financing”[Title/Abstract] OR “health care financing”[Title/Abstract] OR “publicly funded”[Title/Abstract] OR “publicly financed”[Title/Abstract] OR “privately funded”[Title/Abstract] OR “privately financed”[Title/Abstract] OR “externally sourced”[Title/Abstract] OR “external source*”[Title/Abstract] OR “external fund*”[Title/Abstract] OR “foreign fund*”[Title/Abstract] OR “health expenditure”[Title/Abstract] OR “healthcare expenditure”[Title/Abstract] OR “health care expenditure”[Title/Abstract] OR “expenditure on health*”[Title/Abstract] OR “health expenses”[Title/Abstract] OR “health spend*”[Title/Abstract] OR “health investment*”[Title/Abstract] OR “health budget*”[Title/Abstract] OR “health fund*”[Title/Abstract] OR “health account*”[Title/Abstract] OR “monetary”[Title/Abstract] OR “financial”[Title/Abstract])) AND ("low-income country"[Title/Abstract] OR "low-income countries"[Title/Abstract] OR "middle-income country"[Title/Abstract] OR "middle-income countries"[Title/Abstract] OR "low and middle income countries"[Title/Abstract] OR "LMIC"[Title/Abstract] OR "developing countr*"[Title/Abstract] OR "developing nation*"[Title/Abstract] OR "developing econom*"[Title/Abstract] OR "less developed countr*"[Title/Abstract] OR "less developed nation*"[Title/Abstract] OR "less developed econom*"[Title/Abstract] OR "less developed countr*"[Title/Abstract] OR "less developed nation*"[Title/Abstract] OR "less developed econom*"[Title/Abstract] OR "under developed countr*"[Title/Abstract] OR "under developed nation*"[Title/Abstract] OR "under developed countr*"[Title/Abstract] OR "under developed nation*"[Title/Abstract] OR "third world nation*"[Title/Abstract] OR "third world nation*"[Title/Abstract] OR "poor countr*"[Title/Abstract] OR "poor nation*"[Title/Abstract] OR "poor econom*"[Title/Abstract] OR "Afghanistan"[Title/Abstract] OR "Bangladesh"[Title/Abstract] OR "Benin"[Title/Abstract] OR "Bhutan"[Title/Abstract] OR "Burkina Faso"[Title/Abstract] OR "Burundi"[Title/Abstract] OR "Cambodia"[Title/Abstract] OR "Cameroon"[Title/Abstract] OR "Central African Republic"[Title/Abstract] OR "Chad"[Title/Abstract] OR "Comoros"[Title/Abstract] OR "Congo"[Title/Abstract] OR "Democratic Republic of Congo"[Title/Abstract] OR "Cote d'Ivoire"[Title/Abstract] OR "Eritrea"[Title/Abstract] OR "Ethiopia"[Title/Abstract] OR "Gambia"[Title/Abstract] OR "Ghana"[Title/Abstract] OR "Guinea"[Title/Abstract] OR "Guinea-Bissau"[Title/Abstract] OR "Haiti"[Title/Abstract] OR "India"[Title/Abstract] OR "Kenya"[Title/Abstract] OR "Kyrgyzstan"[Title/Abstract] OR "Lao"[Title/Abstract] OR "Lesotho"[Title/Abstract] OR "Liberia"[Title/Abstract] OR "Madagascar"[Title/Abstract] OR "Malawi"[Title/Abstract] OR "Mali"[Title/Abstract] OR "Mauritania"[Title/Abstract] OR "Moldova"[Title/Abstract] OR "Mongolia"[Title/Abstract] OR "Mozambique"[Title/Abstract] OR "Myanmar"[Title/Abstract] OR "Nepal"[Title/Abstract] OR "Nicaragua"[Title/Abstract] OR "Niger"[Title/Abstract] OR "Nigeria"[Title/Abstract] OR "North Korea"[Title/Abstract] OR "Pakistan"[Title/Abstract] OR "Papua New Guinea"[Title/Abstract] OR "Rwanda"[Title/Abstract] OR "Sao Tome and Principe"[Title/Abstract] OR "Senegal"[Title/Abstract] OR "Sierra Leone"[Title/Abstract] OR "Solomon Islands"[Title/Abstract] OR "Somalia"[Title/Abstract] OR "Sudan"[Title/Abstract] OR "Tajikistan"[Title/Abstract] OR "Tanzania"[Title/Abstract] OR "Timor-Leste"[Title/Abstract] OR "Togo"[Title/Abstract] OR "Uganda"[Title/Abstract] OR "Uzbekistan"[Title/Abstract] OR "Vietnam"[Title/Abstract] OR "Yemen"[Title/Abstract] OR "Zaire"[Title/Abstract] OR "Zambia"[Title/Abstract] OR "Zimbabwe"[Title/Abstract] OR "Albania"[Title/Abstract] OR "Algeria"[Title/Abstract] OR "Angola"[Title/Abstract] OR "Armenia"[Title/Abstract] OR "Azerbaijan"[Title/Abstract] OR "Belarus"[Title/Abstract] OR "Bolivia"[Title/Abstract] OR "Bosnia and Herzegovina"[Title/Abstract] OR "Brazil"[Title/Abstract] OR "Bulgaria"[Title/Abstract] OR "Cape Verde"[Title/Abstract] OR "China"[Title/Abstract] OR "Colombia"[Title/Abstract] OR "Cuba"[Title/Abstract] OR "Djibouti"[Title/Abstract] OR "Dominican Republic"[Title/Abstract] OR "Ecuador"[Title/Abstract] OR "Egypt"[Title/Abstract] OR "El Salvador"[Title/Abstract] OR "Fiji"[Title/Abstract] OR "Georgia"[Title/Abstract] OR "Guatemala"[Title/Abstract] OR "Guyana"[Title/Abstract] OR "Honduras"[Title/Abstract] OR "Indonesia"[Title/Abstract] OR "Iran"[Title/Abstract] OR "Iraq"[Title/Abstract] OR "Jamaica"[Title/Abstract] OR "Jordan"[Title/Abstract] OR "Kazakhstan"[Title/Abstract] OR "Kiribati"[Title/Abstract] OR "Macedonia"[Title/Abstract] OR "Yugoslav"[Title/Abstract] OR "Maldives"[Title/Abstract] OR "Marshall Islands"[Title/Abstract] OR "Micronesia"[Title/Abstract] OR "Morocco"[Title/Abstract] OR "Namibia"[Title/Abstract] OR "Paraguay"[Title/Abstract] OR "Peru"[Title/Abstract] OR "Philippines"[Title/Abstract] OR "Romania"[Title/Abstract] OR "Samoa"[Title/Abstract] OR "Serbia and Montenegro"[Title/Abstract] OR "Sri Lanka"[Title/Abstract] OR "Suriname"[Title/Abstract] OR "Swaziland"[Title/Abstract] OR "Syrian Arab Republic"[Title/Abstract] OR "Syria"[Title/Abstract] OR "Thailand"[Title/Abstract] OR "Tonga"[Title/Abstract] OR "Tunisia"[Title/Abstract] OR "Turkmenistan"[Title/Abstract] OR "Ukraine"[Title/Abstract] OR "Vanuatu"[Title/Abstract] OR "West Bank and Gaza"[Title/Abstract] OR "American Samoa"[Title/Abstract] OR "Antigua and Barbuda"[Title/Abstract] OR "Argentina"[Title/Abstract] OR "Barbados"[Title/Abstract] OR "Belize"[Title/Abstract] OR "Botswana"[Title/Abstract] OR "Chile"[Title/Abstract] OR "Costa Rica"[Title/Abstract] OR "Croatia"[Title/Abstract] OR "czech republic"[Title/Abstract] OR "Dominica"[Title/Abstract] OR "Equatorial Guinea"[Title/Abstract] OR "Estonia"[Title/Abstract] OR "Gabon"[Title/Abstract] OR "Grenada"[Title/Abstract] OR "Hungary"[Title/Abstract] OR "Latvia"[Title/Abstract] OR "Lebanon"[Title/Abstract] OR "Libya"[Title/Abstract] OR "Lithuania"[Title/Abstract] OR "Malaysia"[Title/Abstract] OR "Mauritius"[Title/Abstract] OR "Mayotte"[Title/Abstract] OR "Mexico"[Title/Abstract] OR "Northern Mariana Islands"[Title/Abstract] OR "Oman"[Title/Abstract] OR "Palau"[Title/Abstract] OR "Panama"[Title/Abstract] OR "Poland"[Title/Abstract] OR "Russia"[Title/Abstract] OR "Russian Federation"[Title/Abstract] OR "Seychelles"[Title/Abstract] OR "Slovak Republic"[Title/Abstract] OR "South Africa"[Title/Abstract] OR "st kitts and nevis"[Title/Abstract] OR "st lucia"[Title/Abstract] OR "st vincent and the grenadines"[Title/Abstract] OR "Trinidad and Tobago"[Title/Abstract] OR "Turkey"[Title/Abstract] OR "Uruguay"[Title/Abstract] OR "Venezuela"[Title/Abstract]) |
| --- |
